# Chinese Herbal Medicine Baoyuan Jiedu Decoction Inhibited Muscle Atrophy of Cancer Cachexia through Atrogin-l and MuRF-1

**DOI:** 10.1155/2017/6268378

**Published:** 2017-02-14

**Authors:** YaNan Zhang, XiaoChun Han, Bing Ouyang, ZhiChun Wu, HuaYun Yu, Yuan Wang, GuoWei Liu, XuMing Ji

**Affiliations:** ^1^College of Traditional Chinese Medicine, Shandong University of Traditional Chinese Medicine, Jinan, Shandong 250355, China; ^2^Shandong Food and Drug Administration, Jinan, Shandong 250014, China; ^3^College of Foreign Language, Shandong University of Traditional Chinese Medicine, Jinan, Shandong 250355, China

## Abstract

The aim of this study is to investigate the mechanisms of Chinese herbal medicine called “Baoyuan Jiedu” (BYJD for short) decoction, improving life quality and preventing muscle atrophy of cancer cachexia model mice. We showed that the effect of BYJD decoction increased body weights of mice and reduced tumor volume and tumor mass. Furthermore, BYJD decoction increased the gastrocnemii mass and the transverse diameter of muscle fiber morphology. Moreover, BYJD reduced the expression of Atrogin-1 and MuRF-1 protein. Collectively, our results show that BYJD decoction improves the life quality of cancer cachexia mice and prevents muscle atrophy by downregulating expression of Atrogin-1 and MuRF-1.

## 1. Introduction

As a major complication of malignant tumor, cancer cachexia is defined as a “complex metabolic syndrome” characterized by systemic inflammation, negative protein, and energy balance and an involuntary loss of lean body mass [1–3]. Cachexia is implicated in up to 40% of all cancer deaths and is directly responsible for an impaired quality of life and increased health care costs [4]. Weight loss is a prominent clinical feature of cancer cachexia [5, 6]. Both the weight loss and the rate of weight loss are well correlated to cancer mortality [7]. Death usually occurs when there is 30% weight loss [8].

The mechanisms of cancer cachexia are uncertain but may relate to an increase in muscle proteolysis and a decrease in protein synthesis driven by activation of the ubiquitin-proteasome [9–11]. During atrophy, Atrogin-1 and MuRF-1 are the crucial muscle-specific ubiquitin ligases that direct the polyubiquitination of proteins to target them for proteolysis by the 26S proteasome [12], shifting gene expression towards a less myogenic phenotype (Atrogin-1, which degrades MyoD) or mediating sarcomeric breakdown (MuRF-1, which degrades myosins) [13], which has vital significance in the research of mechanism of muscular atrophy of cancer cachexia.

Although our understanding of cancer cachexia has improved dramatically in the past few years, guidelines for the prevention and treatment of cancer-related cachexia are lacking [14]. New treatments with myostatin inhibitors, thalidomide, selective COX-2 inhibitors, ghrelin mimetics, and selective androgen receptor modulators have shown promising results, but their efficacy needs to be confirmed in clinical trials that are at present testing multimodal interventions against cancer cachexia [15, 16]. In contrast, traditional Chinese medicine (TCM) especially for the herbal medicine has long been used for prolonging the survival of patients with malignant tumor by detoxication or nourishing the qi and blood, which may potentially stimulate the immune function. Although the mechanisms of how the herbs demonstrate these effects are unclear and remain to be elucidated, they deserve further studies as new potential therapy agents for cancer cachexia prevention and treatment [17].

BYJD decoction, the composition of which is* Astragalus*,* Ginseng*,* Aconite* root, Honeysuckle,* Angelica*, and Liquiritiae radix, is widely applied clinically in China [18]. Several case reports have demonstrated the safety and efficacy of this decoction in improving quality of life of cancer patients with cachexia. To date, there has been no research investigating the effects of BYJD decoction in improving quality of life and life span associated with cachexia. Therefore, the aims of this research were to (1) investigate the effect of BYJD decoction on inhibiting muscle atrophy and improving quality of life of the mouse model with cancer cachexia and to (2) explore the potential molecular mechanism under the influence of the study intervention.

## 2. Materials and Methods

### 2.1. In Vivo Lewis Lung Carcinoma- (LLC-) Induced Cancer Cachexia Mouse

#### 2.1.1. Experimental Drugs

The Chinese herbal medicine BYJD decoction prescribed by Dr. Feng Li in Shandong University of Traditional Chinese Medicine was provided by Ji'nan Jianlian Medicine Co., Ltd. (Jinan, China). BYJD decoction is composed of* Astragalus* 18 g,* Ginseng* 9 g,* Aconite* root 9 g, Honeysuckle 12 g,* Angelica* 15 g, and Licorice 6 g. The calculated clinical dosages are based on the concentrations in the total crude drug (g) over TCM decoction volume rather than the actual water concentration. And water decoction was concentrated to 1.15 g crude drug/mL, with cold storage at 4°C. Medroxyprogesterone acetate (MPA) was diluted with distilled water and the concentration was 10 mg/mL.

#### 2.1.2. Animals and Tumor Implantation

Lewis lung cancer mice were originally obtained from the Cancer Institute and Hospital, Chinese Academy of Medical Sciences (CAMS). Adult (age: 5 weeks; weight: 20 ± 2 g) C57BL/6 male mice, obtained from the Beijing Weitong Lihua Experimental Animal Technology Co., Ltd., were individually housed and acclimated to their cages and human handling for 1 week before the experiments, in a 12 h light : 12 h dark cycle (lights on at 07:00 h), under controlled temperature conditions (23 ± 1°C), receiving water and food (Animal Experimental Center of Shandong University of Traditional Chinese Medicine) ad libitum. All experiments were conducted with the approval of the Institutional Animal Care and Use Committee and were in compliance with the National Institutes of Health Guidelines for Use and Care of Laboratory Animals.

The 2 Lewis tumor-bearing mice were sacrificed; then the tumor blocks were removed under sterile conditions and made into cell suspension. For the Lewis lung carcinoma- (LLC-) induced cancer cachexia model, 100 mcl (1 × 10^6^ cells) of LLC cells was injected subcutaneously into the right armpit of the mice. Mice of normal control group (A) received saline injections on the same day of tumor inoculation. After tumor inoculation, mice were randomly divided into 4 groups: model control group (B), BYJD treatment group (C), BYJD prevention group (D), and MPA group (E) (*n* = 8).

On the same day of tumor inoculation, mice of BYJD prevention group were given BYJD decoction (0.2 mL/10 g per mouse via gavage, based on the 23 g/kg, 20 g average mouse weight); the other groups were filled with equal doses of normal saline. Cachexia was followed by recording body weight that was plotted as the mean (±SE) against days after tumor transplantation. When the body weight of model mice had dropped to 10% of the healthy controls, this suggested that the cancer cachexia model was successful.

After entering the cachexia period, the mice of BYJD treatment group were given BYJD decoction concentrated liquid by gavage, while the mice of normal group and model control group (B) were given equal doses of normal saline by gavage; the mice of MPA group were given MPA for 15 days. The calculated formula between human and mouse according to the body surface area is mouse dose (g/kg) = human dose (g/kg) × 3/37.

The body weights of the mice were measured once a day. All mice were euthanized on day 30, and tumors were removed and weighted. Tumor volume measurements were calculated using the formula for an oblong sphere: volume = 1/2 × width^2^ × length.

#### 2.1.3. Muscle Fiber Weights and Transverse Diameter Measurements

After mice were sacrificed, gastrocnemii were collected and weighed. 8 um deep cross-sectional tissue sections from the gastrocnemii were fixed in 4% paraformaldehyde, stained with hematoxylin and eosin solutions, and digitally imaged with a microscope (Olympus). For each muscle, at least 8 randomly selected 40x magnification images were quantified with scientific imaging software (ImageJ).

#### 2.1.4. Immunohistochemistry

Paraffin sections were blocked by a blocking buffer for 1 hour at room temperature and stained with a specific primary antibody for 24 hours. The primary antibody was washed using PBS. The sections then were stained with a specific secondary antibody for 24 hours at room temperature and washed with PBS. The primary antibodies used here are listed as follows: Atrogin-1 (1 : 200; Rabbit Polyclonal, lot number: AP2041, ECM Biosciences) and MuRF-1 (1 : 100; Rabbit Polyclonal, lot number: MP3401, ECM Biosciences).

### 2.2. In Vitro Malnutrition Model of Skeletal Muscle Cell by TNF-*α*

#### 2.2.1. Drug Contained Serum Preparation

The rats were randomly divided into medicated serum group and normal serum group. For the medicated serum group, rats were given BYJD decoction (41 g/kg) by gavage, while rats of the normal group were given the same volume of saline 2 times per day for 3 days. Rats were fasted 12 h after the last administration and then given BYJD decoction 1 d dosage. Blood was taken after 1 h and inactivated at 63.5°C and made into freeze-dried powder stored at −70°C.

#### 2.2.2. High Performance Liquid Chromatography (HPLC) Analysis

We performed the initial batch to batch consistency studies using HPLC. Briefly, the plasma sample purification (100 *μ*L) was limited to protein precipitation with methanol (200 *μ*L), and centrifuged supernatant was obtained. Chromatographic conditions were as follows: ginsenoside Rb1 : acetonitrile-0.1% phosphoric acid (32 : 68); chlorogenic acid : acetonitrile-0.4% phosphoric acid (13 : 87); ferulic acid : acetonitrile −0.085% phosphoric acid (17 : 83); aconitine : methanol-water (56 : 44). Column temperature was as follows: ginsenoside Rb1, 40°C, chlorogenic acid, 25°C, ferulic acid, 35°C, and aconitine, 35°C. Wavelength was as follows: ginsenoside Rb1, 203 nm, chlorogenic acid, 327 nm, ferulic acid, 316 nm, and aconitine, 240 nm. Flow rate was 1 mL/min, with sample volume of 20 *μ*L.

#### 2.2.3. Cell Culture and Myotube Transverse Diameter Measurement

C2C12 (GNM26, the Chinese Academy of Sciences cell bank) myoblasts were maintained in Dulbecco's modified Eagle medium (DMEM) (Gibco Company, USA) with 10% foetal bovine serum (Gibco Company, USA), penicillin (200 units/mL), and streptomycin (50 *μ*g/mL) (Invitrogen Company, USA). At 90% confluence, the media were changed to DMEM plus 2% horse serum (HyClone Company, USA) to induce myotube formation. Cells were treated with BYJD decoction medicated serum on day 3 when myotube formed and were harvested 24 h later.

C2C12 cells were randomly divided into 3 groups: normal control group (addition of 0.5% horse serum), TNF-*α* intervention group (addition of 0.5% horse serum and 10 ng/mL TNF-*α*), and medicated serum group (addition of 10% BYJD decoction medicated serum, 0.5% horse serum, and 10 ng/mL TNF-*α*).

Cells were cultured after 72 hours; at least 10 randomly selected 40x magnification images were quantified with scientific imaging software (ImageJ). Each myotube diameter was measured for 3 times and then the results were averaged.

### 2.3. Western Blotting

For Western blotting, total proteins from gastrocnemii in vivo and C2C12 cells in vitro were isolated by homogenization in RIPA buffer with complete protease inhibitor cocktail and phosphatase inhibitor cocktail. Protein quantification was determined by the Bradford protein assay, and samples were equally loaded on 10% polyacrylamide gels electrophoresed at 200 V, electrotransferred to PVDF membranes, and probed with antibodies to Atrogin-1 (1 : 1000; Rabbit Polyclonal, lot number: AP2041, ECM Biosciences) and MuRF-1 (1 : 1000; Rabbit Polyclonal, lot number: MP3401, ECM Biosciences). The images were scanned, and blots were quantified by densitometry using scientific imaging software (ImageJ) and normalized to loading controls.

### 2.4. Statistical Analysis

All data were expressed as means ± SD of three independent experiments. Statistical significance was determined by one-way ANOVA followed by Bonferroni's multiple comparison test (SPSS 22.0). Differences were considered statistically significant when *P* < 0.05.

## 3. Results

### 3.1. BYJD Decoction Prevented LLC-Induced Cachexia

**Figure 1:**
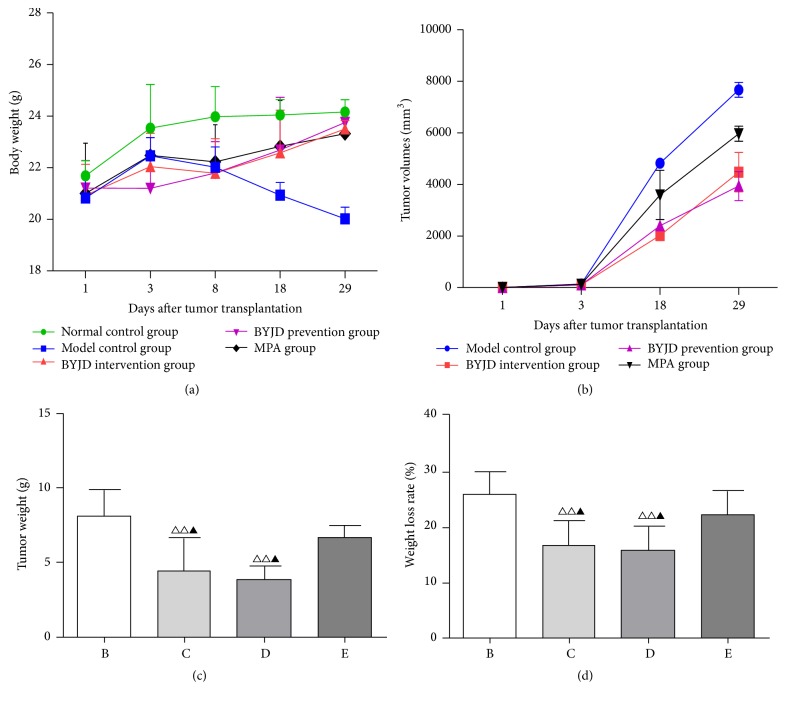
BYJD decoction and MPA improve LLC-induced cachexia. (a) Changes in body weight in normal group, model control group, BYJD treatment group, BYJD prevention group, and MPA group were measured on days 1, 3, 8, 18, and 29. (b) Changes in tumor volume in normal group, model control group, BYJD intervention group, BYJD prevention group, and MPA group were measured on days 1, 3, 8, 18, and 29. (c) Tumor weight in model group (B), BYJD treatment group (C), BYJD prevention group (D), and MPA group (E) was measured on day 29 after tumor cells inoculation. (d) Weight loss rates were measured in model control group (b), BYJD treatment group (c), BYJD prevention group (d), and MPA group (e). Values are expressed as mean ± SD of eight mice. ^△△^*P* < 0.01 compared with the model control group; ^▲^*P* < 0.05 compared with the MPA group.

The LLC transplanted in mice formed a rapidly-growing tumor causing progressive body weight loss (BWL), typical of the cachectic syndrome. As shown in Figure 1(a), all LLC-bearing mice began to lose weight (a hallmark of cachexia) 3 days after subcutaneous tumor transplantation. Until 8 days after tumor transplantation, the LLC-bearing mice developed cachexia (BWL > 10%). Compared with model control mice, LLC induced a significant decrease in body weight, whereas BYJD decoction and MPA administration prevented these changes within 29 days after tumor transplantation.

We also tested therapeutic efficacy of BYJD decoction on the growth of tumor. We noticed a significant reduction of the tumor weight and sizes observed in the BYJD decoction treatment group compared to the model control group (Figures 1(b) and 1(c)). We found that, compared to the model control group, the BYJD decoction treated mice and BYJD decoction prevented mice showed a significant growth-inhibitory effect, and the effect was better than MPA (Figure 1(d)).

### 3.2. BYJD Decoction Prevents LLC-Induced Muscle Wasting

**Figure 2:**
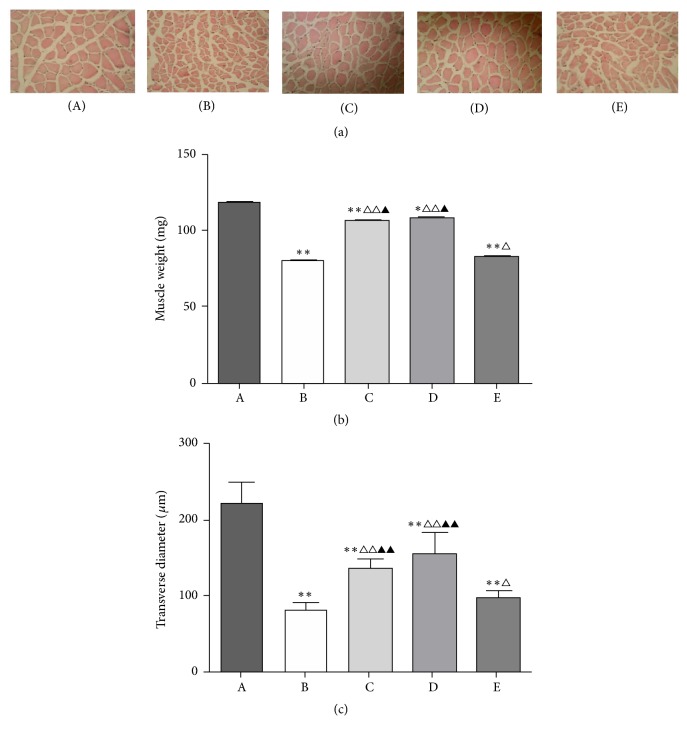
BYJD decoction and MPA prevent LLC-induced muscle wasting. (a) Representative histological images of gastrocnemii fiber (HE ×40). Scale bars = 167 *μ*m. (b) Changes of gastrocnemii weight in normal group (A), model control group (B), BYJD treatment group (C), BYJD prevention group (D), and MPA group (E). (c) Changes of the transverse diameter in normal group (A), model control group (B), BYJD treatment group (C), BYJD prevention group (d), and MPA group (e). Values are expressed as mean ± SD of eight mice. ^*∗*^*P* < 0.05 and ^*∗∗*^*P* < 0.01, compared with normal control group; ^△^*P* < 0.05 and ^△△^*P* < 0.01, compared with the model control group; ^▲^*P* < 0.05 and ^▲▲^*P* < 0.01, compared with the MPA group.

One of the key features of cancer-induced wasting syndrome is the loss of skeletal muscle mass; to test whether cachexia was accompanied by skeletal muscle atrophy and whether BYJD decoction prevented this, mice bearing subcutaneous LLC were treated with BYJD decoction or MPA and muscle wasting was analyzed at autopsy. After the mice were sacrificed, gastrocnemii were collected and weighed. The weight of gastrocnemii from model control group decreased (Figure 2(b)). Strikingly, compared to the model control group, the mean weight of gastrocnemii from BYJD treatment group, BYJD prevention group, and MPA group mice increased, indicating that BYJD decoction treatment protects from LLC-induced muscle wasting.

Moreover, the transverse diameter of the gastrocnemii myofibers showed a significant reduction between model control group and normal control group which was partial but restored upon BYJD treatment group, BYJD prevention group, and MPA group (Figures 2(a) and 2(c)).

### 3.3. BYJD Decoction Prevents Atrogin-1 and MuRF-1 Activation in Muscles of LLC-Bearing Mice

**Figure 3:**
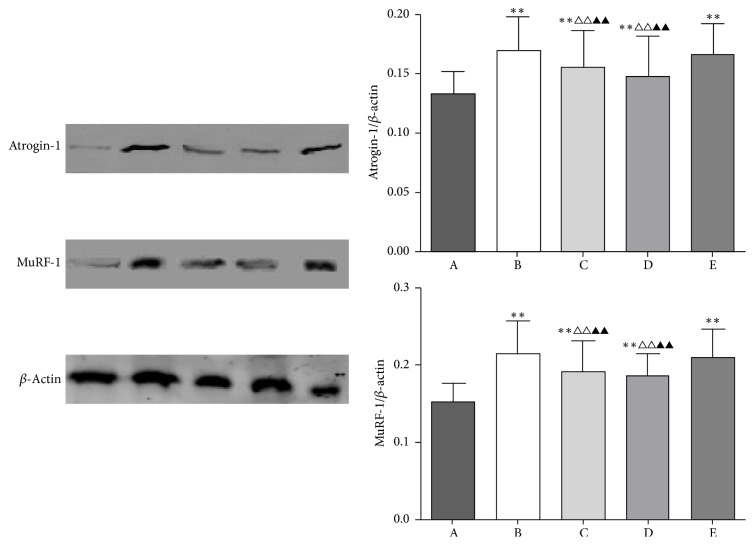
Western blot analysis for expressions of Atrogin-1, MuRF-1, and *β*-actin in gastrocnemii from normal group (A), model control group (B), BYJD treatment group (C), BYJD prevention group (D), and MPA group (E). The graph represents relative densitometric intensity of each band normalized to *β*-actin. Values are expressed as mean ± SD of eight mice. ^*∗∗*^*P* < 0.01 compared with normal control group; ^△△^*P* < 0.01 compared with the model control group; ^▲▲^*P* < 0.01 compared with the MPA group.

**Figure 4:**
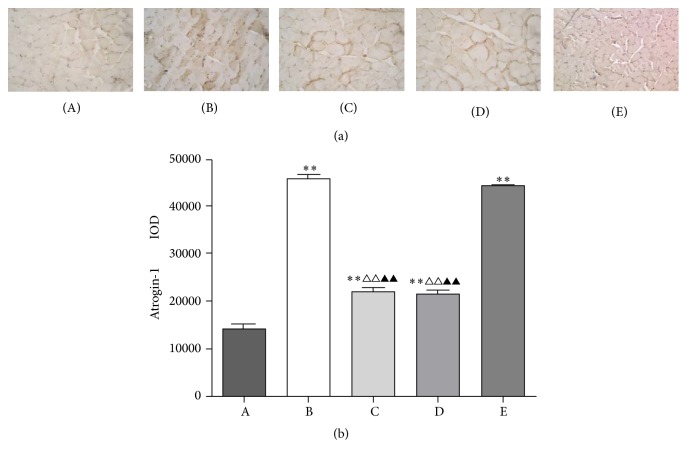
Immunohistochemistry of gastrocnemii from normal group (A), model control group (B), BYJD treatment group (C), BYJD prevention group (D), and MPA group (E), where protein expressions are shown for Atrogin-1. (b) Values are expressed as mean ± SD of eight mice. ^*∗∗*^*P* < 0.01 compared with normal control group; ^△△^*P* < 0.01 compared with the model control group; ^▲▲^*P* < 0.01 compared with the MPA group.

**Figure 5:**
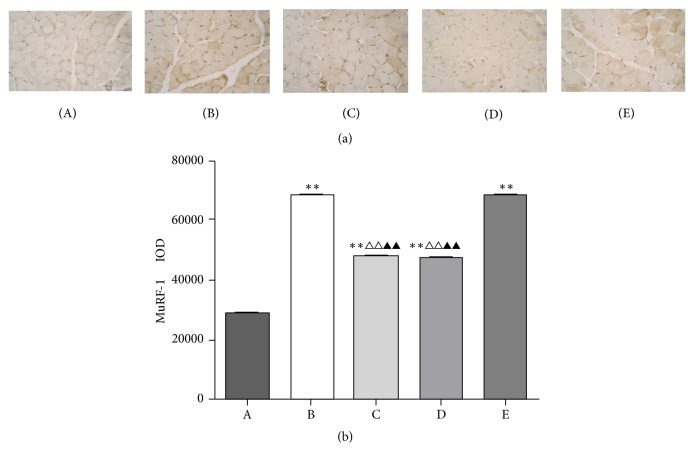
Immunohistochemistry of gastrocnemii from normal group (A), model control group (B), BYJD treatment group (C), BYJD prevention group (D), and MPA group (E), where protein expressions are shown for MuRF-1. (b) Values are expressed as mean ± SD of eight mice. ^*∗∗*^*P* < 0.01 compared with normal control group; ^△△^*P* < 0.01 compared with the model control group; ^▲▲^*P* < 0.01 compared with the MPA group.

Loss of skeletal muscle mass is generally due to reduced protein synthesis, increased degradation, or a relative imbalance of the two [8, 9]. Atrogin-1 and MuRF-1, two muscle-restricted ubiquitin ligases involved in the accelerated protein degradation during various kinds of muscle atrophy [10], were found to be highly upregulated in gastrocnemii from model control group mice, while BYJD decoction prevention and treatment were able to prevent upregulation of Atrogin-1 and MuRF-1. MPA treatment led to downregulation of Atrogin-1 and MuRF-1, although the effect was less evident (Figures 3, 4, and 5). Overall, these data indicate that LLC-bearing mice offer a unique model to test drugs against cancer cachexia and that BYJD decoction can prevent cachexia in vivo by lowering protein catabolism through inhibition of Atrogin-1 and MuRF-1 activation at least in muscles.

### 3.4. Effect of BYJD Decoction on Myotube Breakdown and Proteolysis Changes Induced by TNF-*α* In Vitro

**Figure 6:**
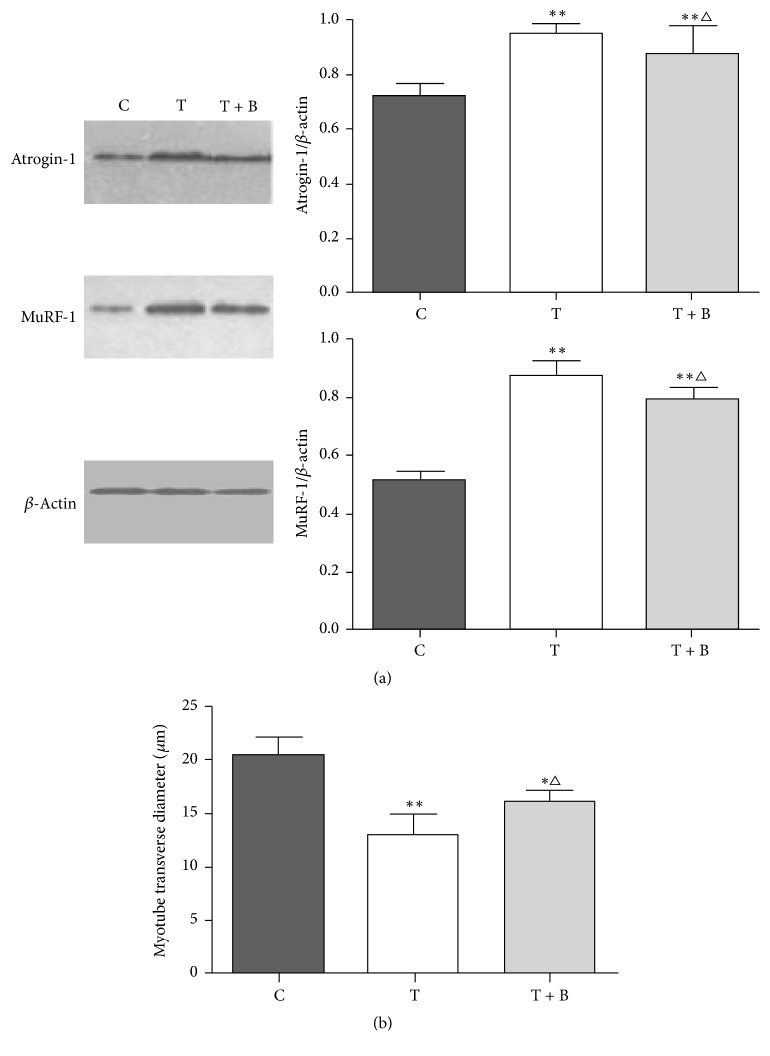
(a) Western blot analysis for expressions of Atrogin-1, MuRF-1, and *β*-actin in C2C12 myotubes normal control group (C), TNF-*α* intervention group (T), and BYJD decoction medicated serum group (T + B). The graph represents relative densitometric intensity of each band normalized to *β*-actin. (b) Changes of myotube transverse diameter in normal control group (C), TNF-*α* intervention group (T), and BYJD decoction medicated serum group (T + B). Values are expressed as mean ± SD of eight mice. ^*∗*^*P* < 0.05 and ^*∗∗*^*P* < 0.01, compared with normal control group; ^△^*P* < 0.05 compared with the TNF-*α* intervention group.

According to HPLC, BYJD water decoction and BYJD decoction medicated serum contain ginsenoside Rb1, chlorogenic acid, ferulic acid, and aconitine. To further characterize the mechanisms mediating the effects of BYJD decoction in cachexia, C2C12 myotubes were treated with BYJD decoction medicated serum. TNF-*α* induced a significant decrease in myotube size and myotube transverse diameter, and these changes were prevented by BYJD decoction medicated serum (Figure 6(b)). TNF-*α* increased the expression of Atrogin-1 and MuRF-1, and these changes were prevented by BYJD decoction medicated serum (Figure 6(a)).

## 4. Discussion

Lung carcinoma is one of the malignancies that mostly cause a cancer-associated systemic syndrome (i.e., cachexia) [19]. At the time of diagnosis, 60% of lung cancer patients have cachexia [20]. Cachexia is a devastating complication of cancer which contributes to a decrease in quality of life and early demise in individuals with this condition [21]. Traditional Chinese medicine (TCM) has significant effect on improving the quality of life of patients with cancer cachexia [22], but the specific mechanism is unclear.

BYJD decoction is composed of* Astragalus*,* Ginseng*,* Aconite* root, Honeysuckle,* Angelica*, and Liquiritiae radix.* Astragalus* is sweet and mild in nature and has the function of reinforcing vital energy and benefiting Yang-qi, especially the qi of spleen and lungs; Ginseng is beneficial to both the spleen and stomach and it assists* Astragalus* in replenishing qi to invigorate the spleen and strengthening muscles;* Aconite* root circulates in the twelve meridians and warms Yang-qi; therefore, it matches with* Astragalus* and* Ginseng* to reinforce both qi and Yang and serves as ministerial drugs; Honeysuckle clears away heat and toxic material, while* Angelica sinensis* reinforces and invigorates blood;* Angelica sinensis* invigorates both qi and blood when it is matched with* Astragalus* and* Ginseng*; meanwhile, it could invigorate blood and erase blood stagnation when it is matched with Honeysuckle; therefore, both Honeysuckle and* Angelica sinensis* serve as adjuvant drugs, directed at erasing stasis and toxin; Liquiritiae radix is used as harmonizing drug to coordinate the drug actions of a prescription and clear away heat and toxic material. BYJD decoction has the function of treating lungs and spleen together, benefiting both qi and Yang, supporting health qi, and resolving toxin.

In this study, we report that treatment with BYJD decoction improves life quality of cancer cachexia of mice bearing the Lewis lung carcinoma, transplanted in the subcutis by blocking the BWL (i.e., muscle wasting) caused by cancer growth. The results indicate that BYJD decoction improves the life quality of cancer cachexia mice. Compared with the model control group, the body weights of mice of BYJD decoction prevention group and BYJD decoction treatment group increased, while the tumor volume and tumor mass reduced and the gastrocnemii mass and the transverse diameter of muscle fiber morphology increased. Compared with MPA group, BYJD decoction prevention group and BYJD decoction treatment group have no significant difference in increasing body weight; however, BYJD decoction has a function of increasing gastrocnemii weight and improving muscular atrophy, which confirms the role of BYJD decoction in improving the life quality and muscle waste of cancer cachexia.

Muscle waste is independently associated with indices of quality of life. The mechanism of skeletal muscle wasting may be attributed to the increase of protein degradation and the synthesis of the protein [23]. Skeletal muscle protein degradation and the activation of ATP ubiquitin-proteasome pathways are closely related [24]. E3 is the key enzyme of UPP activity of cancer cachexia and it determines the specificity and rate of UPP system [25, 26]. The activation of constituents such as Atrogin-1 and MuRF-1 of E3 accelerates the substrate ubiquitination, improves protein degradation in proteasomes, and enhances the atrophy and loss of lean body mass, such as skeletal muscle, which finally leads to cancer cachexia [27]. Our data demonstrated that Atrogin-1 and MuRF-1 are closely related to muscular atrophy. Of note, BYJD decoction can reduce Atrogin-1 and MuRF-1 protein expression in the skeletal muscle of cancer cachexia. Our results show that BYJD decoction inhibits gastrocnemii atrophy through reducing Atrogin-1 and MuRF-1 protein expression.

In the study, we demonstrated that the curative effects were similar in mice of BYJD decoction prevention group and BYJD decoction treatment group. BYJD decoction was used to prevent cancer cachexia on the same day of tumor inoculation which could delay the appearance of cachexia signs and slowed rates of weight loss and tumor mass growth. It reflects the thought of “preventing measures taken before the occurrence of disease” in Huangdi's Internal Classic.

We also studied a model of malnutrition model of skeletal muscle cell by TNF-*α* in C2C12 cells to determine if the effects of BYJD decoction were due to a direct effect on muscle cells. Administration of TNF-*α* to C2C12 cells induced myotube atrophy, and this was associated with activation of the ubiquitin ligases Atrogin-1 and MuRF-1; meanwhile BYJD decoction medicated serum was able to inhibit myotube atrophy and prevent upregulation of Atrogin-1 and MuRF-1, as we had demonstrated in vivo. This suggests that BYJD decoction exerts at least part of its effects directly on muscle.

Overall, we show that BYJD decoction prevents BWL and preserves muscle by inhibiting upregulation of Atrogin-1 and MuRF-1. Although our results encourage regarding BYJD decoction as therapeutic option for cancer cachexia patients, the mechanism involved in the effect of BYJD decoction in human needs to be further determined in the future.

## 5. Conclusion

Taking all data together, we conclude that the cachectic state developed by tumor effects, mainly impairing the muscle tissue, as a consequence of higher activity of the ubiquitin-proteasome pathway, can be minimized under BYJD decoction treatment as this treatment ameliorated chemical body composition, reducing the ubiquitin-proteasome pathway, which decreased the protein waste. In summary, BYJD decoction remains a promising tool in cancer studies and how it could attenuate cachexia in tumor-bearing animals is a subject for further research.
